# Higher phosphate concentrations as in aqueous humor of diabetic patients increase intraocular lens calcification

**DOI:** 10.1186/s12886-024-03553-z

**Published:** 2024-08-23

**Authors:** Rebecca Buhl, Timur Mert Yildirim, Sonja Katrin Schickhardt, Leoni Britz, Ingo Lieberwirth, Gerd Uwe Auffarth, Ramin Khoramnia

**Affiliations:** 1 The David J. Apple International Laboratory for Ocular Pathology, Department of Ophthalmology, University Hospital of Heidelberg, Heidelberg, Germany; 2 Department of Physical Chemistry of Polymers, Max Planck Institute for Polymer Research, Mainz, Germany; 3 Heidelberg University , Heidelberg, Germany

**Keywords:** IOL Calcification, Diabetes, Proliferative diabetic retinopathy, Aqueous humor, Phosphate concentration

## Abstract

**Background:**

Clinical evidence suggests an association between phosphate concentrations in aqueous humor and the risk of intraocular lens (IOL) calcification. To test this hypothesis the influence of different phosphate concentrations on IOL calcification was evaluated in an in vitro electrophoresis model.

**Methods:**

20 IOLs of two hydrophilic IOL models (CT Spheris 204, Zeiss; Lentis L-313, Oculentis) and one hydrophobic control IOL model (Clareon CNA0T0, Alcon) were exposed to physiologic and elevated phosphate concentrations, similar to diabetic aqueous humor. IOL calcification was analyzed by alizarin red staining, von Kossa staining, scanning electron microscopy, energy dispersive x-ray spectroscopy and transmission electron microscopy with electron diffraction.

**Results:**

Higher phosphate concentrations were associated with IOL calcification. Analyses of IOL surfaces and cross-sections documented calcification in no CT Spheris and 4 Lentis IOLs following exposure to 10 mM Na_2_HPO_4_, compared with 7 and 11 positive analyses following exposure to 14 mM Na_2_HPO_4_, respectively. Furthermore, a clear association between IOL calcification and the duration of electrophoresis was demonstrated, confirming increased phosphate concentrations and duration of exposure as risk factors of IOL calcification.

**Conclusions:**

Findings suggest that higher phosphate concentrations in aqueous humor, as seen in diabetic patients, contribute to an increased IOL calcification risk, potentially explaining clinical observations showing an increased risk of IOL calcification in patients with diabetes.

## Background

Intraocular lens (IOL) calcification is a form of opacification in which the precipitation of calcium and phosphate from aqueous humor occurs within or at the surface of hydrophilic acrylic IOLs, leading to the formation of calcium phosphate crystals [1, 2]. The crystal formation within the IOL causes a range of symptoms including glare and other forms of visual impairment, all with a major impact on patients’ vision [3–5]. The only possible intervention to restore patients’ vision with calcified hydrophilic IOLs is explantation and reimplantation of a new IOL. IOL calcification has become a major reason for explantation in recent years [3]. This is even more clinically relevant as IOL exchange is associated with a high intraoperative complication rate [6, 7].

IOL calcification can be clinically categorized into primary, secondary, and pseudo-calcification. As pseudo-calcification is defined as a misdiagnosis; mostly due to the misconception of posterior capsular opacification as IOL calcification or a false-positive histological staining, only the division into primary and secondary calcification is clinically relevant [8]. Primary calcification is thought to be associated with the IOL itself, usually also occurring in eyes without comorbidities [3, 9–11]. The underlying causes lie mainly in the chemistry of the IOL polymers or faulty manufacturing and usually affect entire batches of IOL models [3, 5, 10, 12]. These issues have been recognized and addressed by manufacturers, potentially making primary calcification less relevant in the future. Secondary IOL calcification, however, attributable to multiple external and patient associated risk factors, is also a major concern following implantation of any hydrophilic IOL, independent of manufacturer or IOL model. Typical factors considered to increase the risk of secondary calcification include patients’ comorbidities such as diabetes, other systemic or ocular diseases, ultimately any condition disrupting the blood-aqueous barrier [13–17]. Changes in the aqueous milieu surrounding the implanted IOL as well as surgical procedures with intraocular injection of gas, air or silicone oil also likely increase the risk of IOL calcification [6, 13, 14, 18–21]. It is important to recognize, however, that whilst triggering factors vary in primary and secondary calcification, the underlying mechanisms and calcification processes initiated in the IOL polymer are most probably identical.

For this reason, as the pathological mechanisms leading to IOL calcification are not yet completely understood, identifying risk factors plays a key role in preventing and reducing IOL calcification. Many case studies suggest diabetes as a major risk factor, pointing to an as yet unproven causal correlation between diabetes and IOL calcification [14, 15, 17, 22–26]. Initial analyses found increased calcium and phosphate concentrations in aqueous humor of diabetic patients, based on findings in 11 eyes [14]. In a larger analysis of 128 patients, 52 diabetics and 76 non-diabetics undergoing cataract surgery, Kim et al. confirmed increased phosphate concentrations in aqueous humor of diabetic patients, showing an increase from the physiologic concentration by a factor of 1.2 in patients with diabetes and of 1.4 in patients with proliferative diabetic retinopathy [15].

**Fig. 1:**
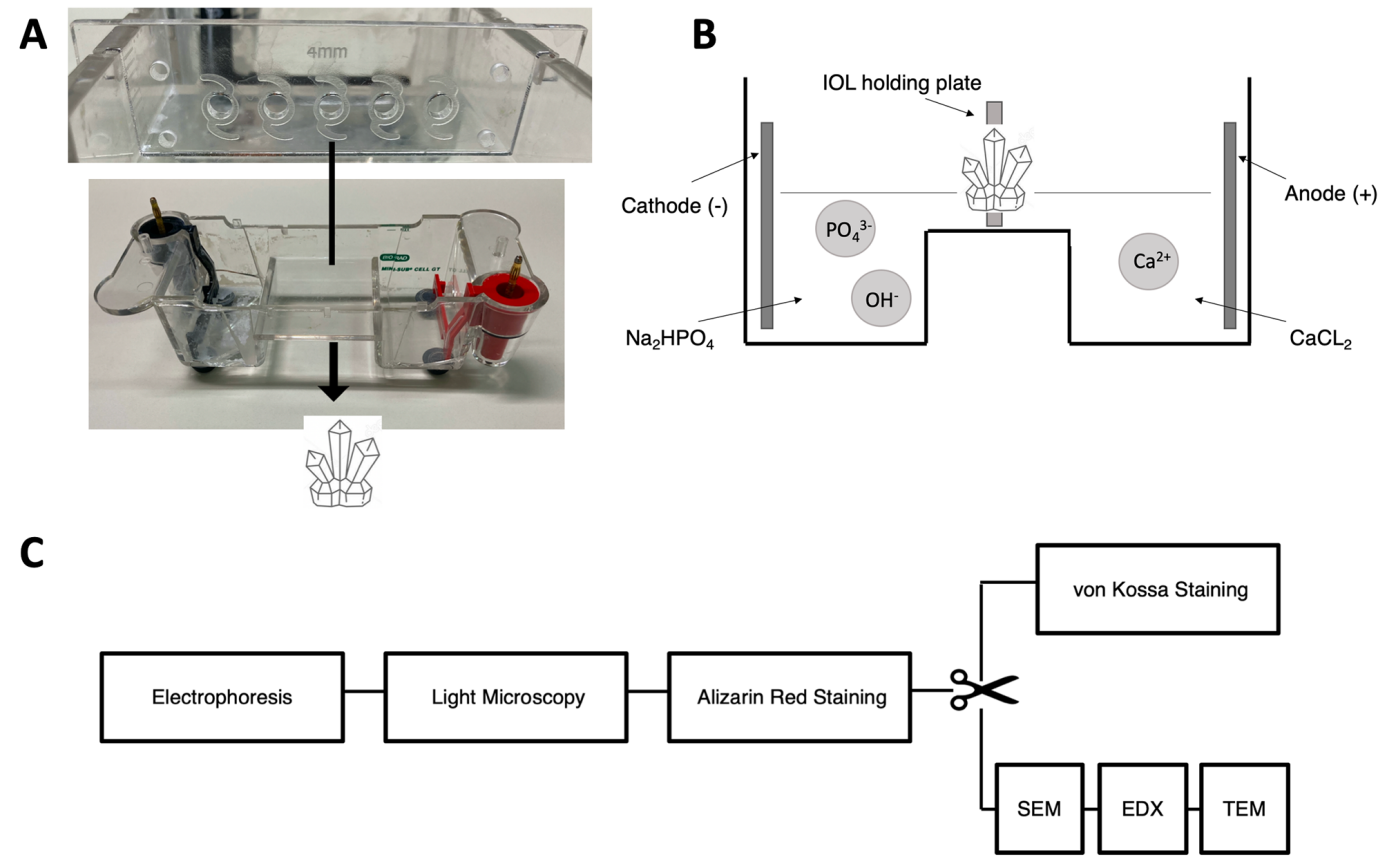
Electrophoresis model and IOL analysis protocol. **a**. Electrophoresis model with the cathode (left) and anode (right) chamber and the IOL holding plate above. **b**. Schematic demonstration of the electrophoresis model with the Na_2_HPO_4_ solution at the cathode (left) and CaCl_2_ solution at the anode (right). The phosphate and calcium ions diffuse to the other side respectively, passing through the IOLs in the IOL holding plate, causing crystal formation and therefore IOL calcification. CaCl_2_: Calcium chloride, Na_2_HPO_4_: Disodium hydrogen phosphate. **c**. Following electrophoresis, the IOLs underwent different analyses as indicated. Gross light microscopy was performed to obtain overview images. Alizarin red staining was used to detect superficial calcium phosphate deposits. Subsequently, the IOL was cut in half and the von Kossa method was used to identify deposits below the IOL polymer surface in cross-sections of one IOL half. SEM and EDX were performed at the Max Planck Institute for polymer research in Mainz (Germany) to detect crystal growth in IOL cross-sections. TEM and ED were performed following EDX, allowing definitive identification of the specific calcium phosphate crystal present. SEM: Scanning electron microscopy, ED: Electron diffraction, EDX: Energy-dispersive X-ray spectroscopy, TEM: Transmission electron microscopy with electron diffraction

Consequently, to test this hypothesis, the present analyses aimed to determine a potential association between the diabetic metabolic pattern in the eye and IOL calcification. An in vitro model recently developed by our group succeeded at replicating IOL calcification using an electrophoresis setup with calcium chloride (CaCl_2_) and disodium hydrogen phosphate (Na_2_HPO_4_) solutions to simulate the mechanisms underlying IOL calcification (Fig. 1A and B) [27–30]. This model is based on counter diffusion of calcium and phosphate ions. When these ions meet within the polymer matrix of the IOLs and their concentrations exceed the solubility product of a calcium phosphate compound, IOL calcification will occur. In the present analyses this model was modified to test how the diabetic metabolic state influences IOL calcification, by applying increased phosphate concentrations, mimicking a diabetic environment in the eye [15]. Furthermore, the correlation between IOL type, phosphate concentration, duration of exposure and extent of IOL calcification was evaluated.

## Methods

### Electrophoresis model set-up

An in vitro electrophoresis model was used to replicate in vivo IOL calcification and to test the influence of the diabetic metabolic state on IOL calcification [27–29]. Physiologic and increased phosphate concentrations in aqueous humor, as seen in patients with diabetes, were applied and simulated in the electrophoresis chamber and subsequent IOL calcification was investigated. Two hydrophilic IOL models (CT Spheris 204 by Zeiss and Lentis^®^L-313 by Oculentis) and one hydrophobic IOL (Clareon^®^IOL CNA0T0) used as a control were exposed to the different phosphate concentrations (Table 1). Following 5–20 h of electrophoresis, the presence of calcium phosphate crystals on the surface and within the IOL polymer was evaluated by 7 specific methods (Fig. 1C).

10 mM CaCl_2_ and two different concentrations of Na_2_HPO_4_ aqueous solutions were placed in the anode and cathode reservoir of the electrophoresis model respectively (Mini-Sub Cell GT Cell horizontal electrophoresis tank) and the IOL, acting as a semi-permeable membrane, were placed in a double-walled IOL-holder of our design (Fig. 1A and B). A rubber seal was used to prevent solution exchange between the anode and cathode reservoir, ensuring that all ion diffusion occurred exclusively through the IOL acting as semi-permeable membrane. Using a platinum electrode (Bio-Rad Laboratories Inc., Hercules, California, USA), a standardized setting of 100 V, 25 mA and 41 W, previously established in our laboratory for the development of in vitro IOL calcification, was applied for five hours, facilitating migration of the ions through the IOL polymer and enabling calcium phosphate precipitation, a prerequisite of hydroxyapatite formation and IOL calcification [30]. The temperature was kept at constant 22 °C. Analysis of human aqueous humor demonstrated an increase of phosphate concentrations in patients with proliferative diabetic retinopathy by a factor of 1.4, so in our experiments both physiologic (10 mM) and diabetic (14 mM) Na_2_HPO_4_ concentrations were used [15]. A 10 mM Tris(hydroxymethyl)aminomethane and 10 mM HCl-buffer were used to adjust all solutions to a pH of 7.4. For every IOL model and every concentration, 4 hydrophilic IOLs and one hydrophobic control IOL were tested. Every 5 h one IOL was removed and the solutions were renewed to maintain stable ion concentrations and standard settings for the remaining IOLs. This was repeated four times, so that the last hydrophilic IOL and control IOL underwent 20 h of electrophoresis, 4 rounds of 5 h (Table 1). A total of 16 hydrophilic IOLs (8 CT Spheris and 8 Oculentis) and 4 hydrophobic control IOLs were tested.

**Table 1:** Overview of IOL models, Na_2_HPO_4_ concentrations and exposure durations tested

Na_2_HPO_4_ Concentration	CT Spheris IOL	Lentis IOL	Clareon control IOL
**10 mM**	5, 10, 15, 20 h	5, 10, 15, 20 h	20 h
**14 mM**	5, 10, 15, 20 h	5, 10, 15, 20 h	20 h

h: hours, mM: millimolar, Na_2_HPO_4_: Disodium hydrogen phosphate

### IOL analysis protocol

**Fig. 2:**
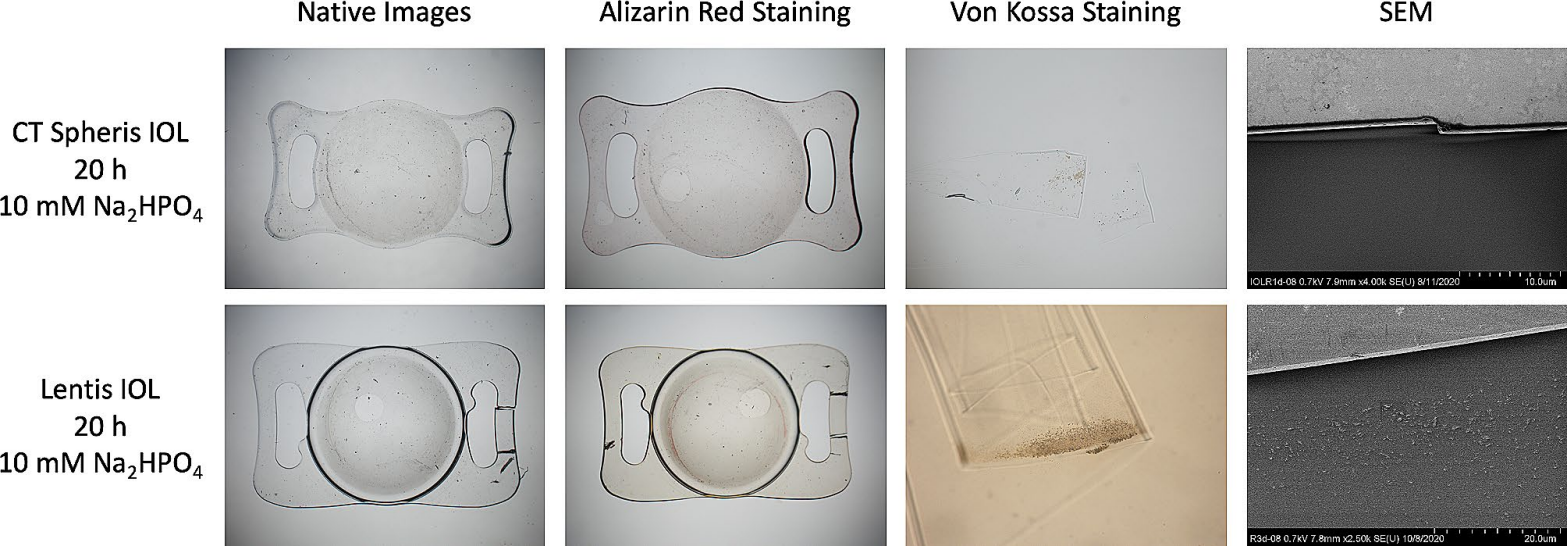
CT Spheris (top row) and Lentis IOL (bottom row) after 20 h exposure to 10 mM Na_2_HPO_4_. Native images show a general overview. Alizarin red staining detects superficial calcium phosphate deposits. Once the IOL has subsequently been halved, von Kossa staining identifies deposits below the IOL polymer surface in cross-sections of the IOL. Similarly to von Kossa staining, SEM identifies crystals below the IOL surface in cross-sections obtained following halving of the IOL (Fig. 4A1). EDX analysis confirmed that the crystals contain calcium and phosphorus. The bottom left scale bars show 0.5 mm. Na_2_HPO_4_: Disodium hydrogen phosphate, EDX: Energy-dispersive X-ray spectroscopy, SEM: Scanning electron microscopy

**Fig. 3:**
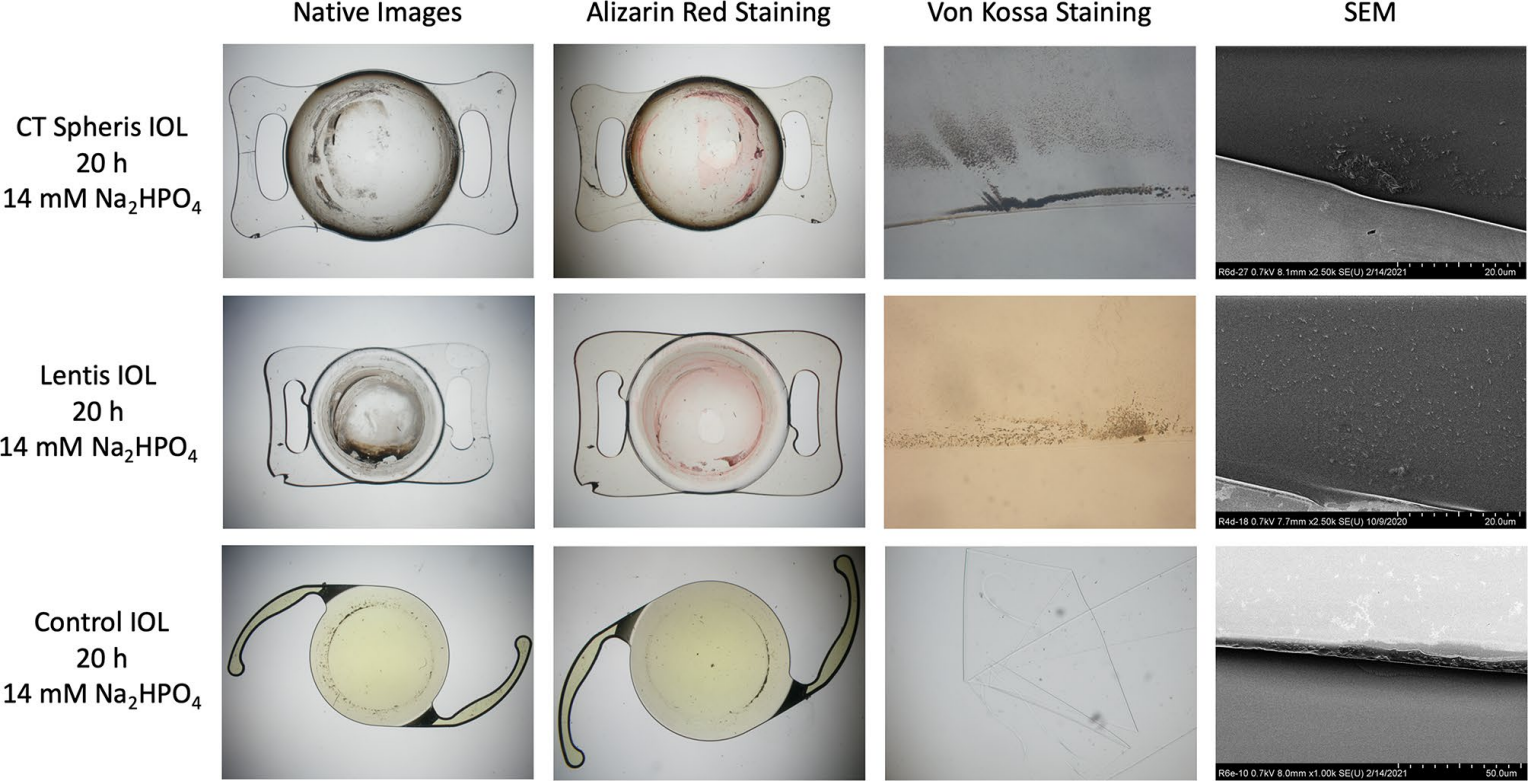
CT Spheris (top row), Lentis (middle row) and Clareon IOL (bottom row) after 20 h exposure to 14 mM Na_2_HPO_4_. Native images show a general overview. Alizarin red staining detects superficial calcium phosphate deposits. Once the IOL has subsequently been halved, von Kossa staining identifies deposits below the IOL polymer surface in cross-sections of the IOL. Similarly to von Kossa staining, SEM identifies crystals below the IOL surface in cross-sections obtained following halving of the IOL (Fig. 4A1). EDX analysis confirmed that the crystals contain calcium and phosphorus. Compared to the CT Spheris (top row) and Lentis (middle row) IOLs, no positive histologic staining occurred in the Clareon control IOL and SEM images obtained of both surfaces and cross-sections of the IOL remained free of any crystal growth. The bottom left scale bars show 0.5 mm. Na_2_HPO_4_: Disodium hydrogen phosphate, EDX: Energy-dispersive X-ray spectroscopy, SEM: Scanning electron microscopy

**Fig. 4:**
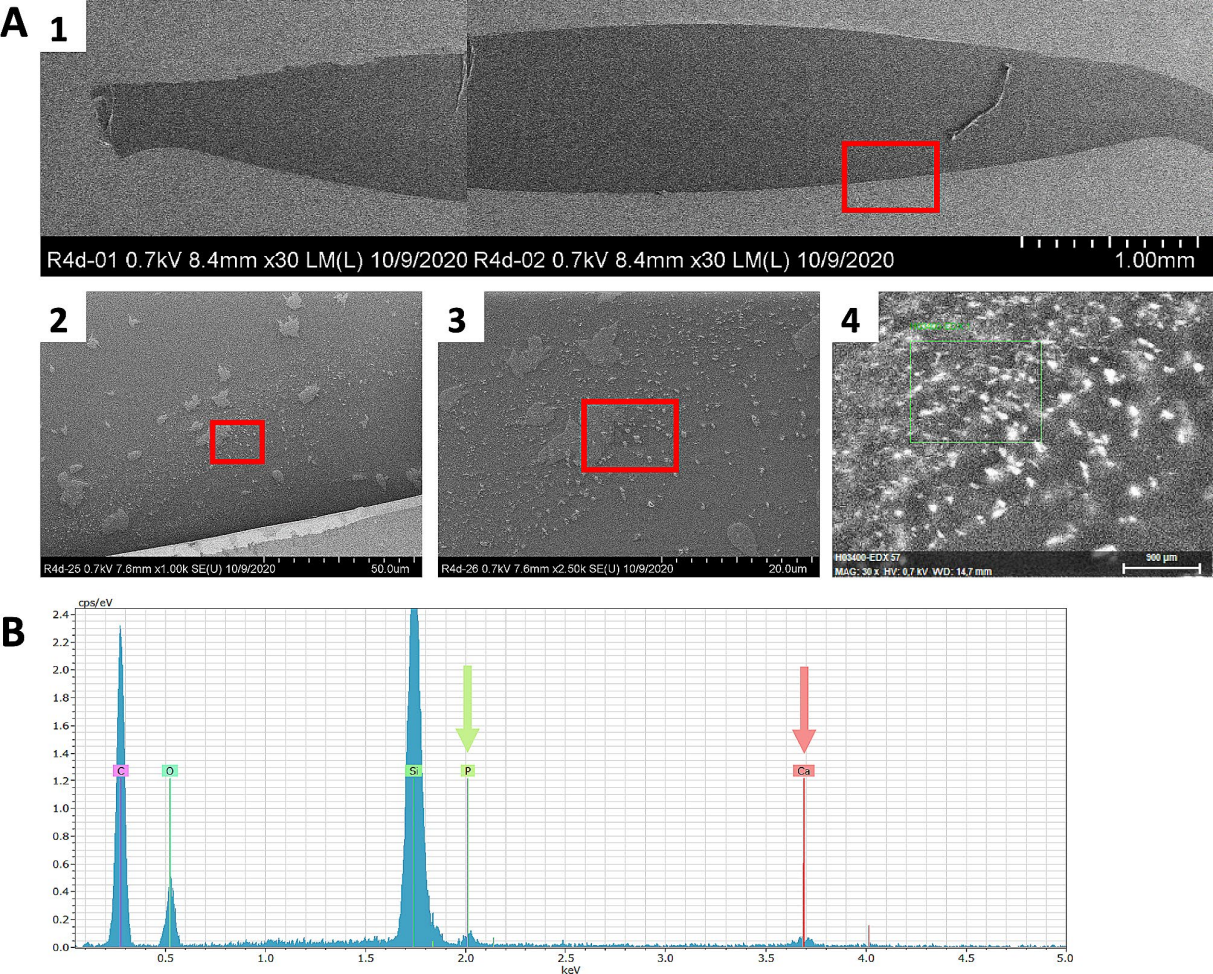
Both SEM overview and magnification as well as EDX microanalysis of crystal growth in the Lentis IOL following 20 h exposure to 14 mM Na_2_HPO_4_. **a**_1_. SEM overview of the investigated IOL cross-section. **a**_2,3_. SEM magnification of the investigated IOL cross-section documenting calcium phosphate crystal growth. **a**_4_. EDX analysis of the precise area indicated. **b**. Analysis of the elements present shows distinctive peaks for calcium (Ca) and phosphorus  (P) and confirms that the crystal contains both elements. The silicon peak (Si) is an artefact created because a silicon wafer is used in the analysis. The peaks for carbon (C) and oxygen (O) are also expected as these are elements contained in hydroxyapatite, the present calcium phosphate salt. Na_2_HPO_4_: Disodium hydrogen phosphate, SEM: Scanning electron microscopy, EDX: Energy-dispersive X-ray spectroscopy

**Fig. 5:**
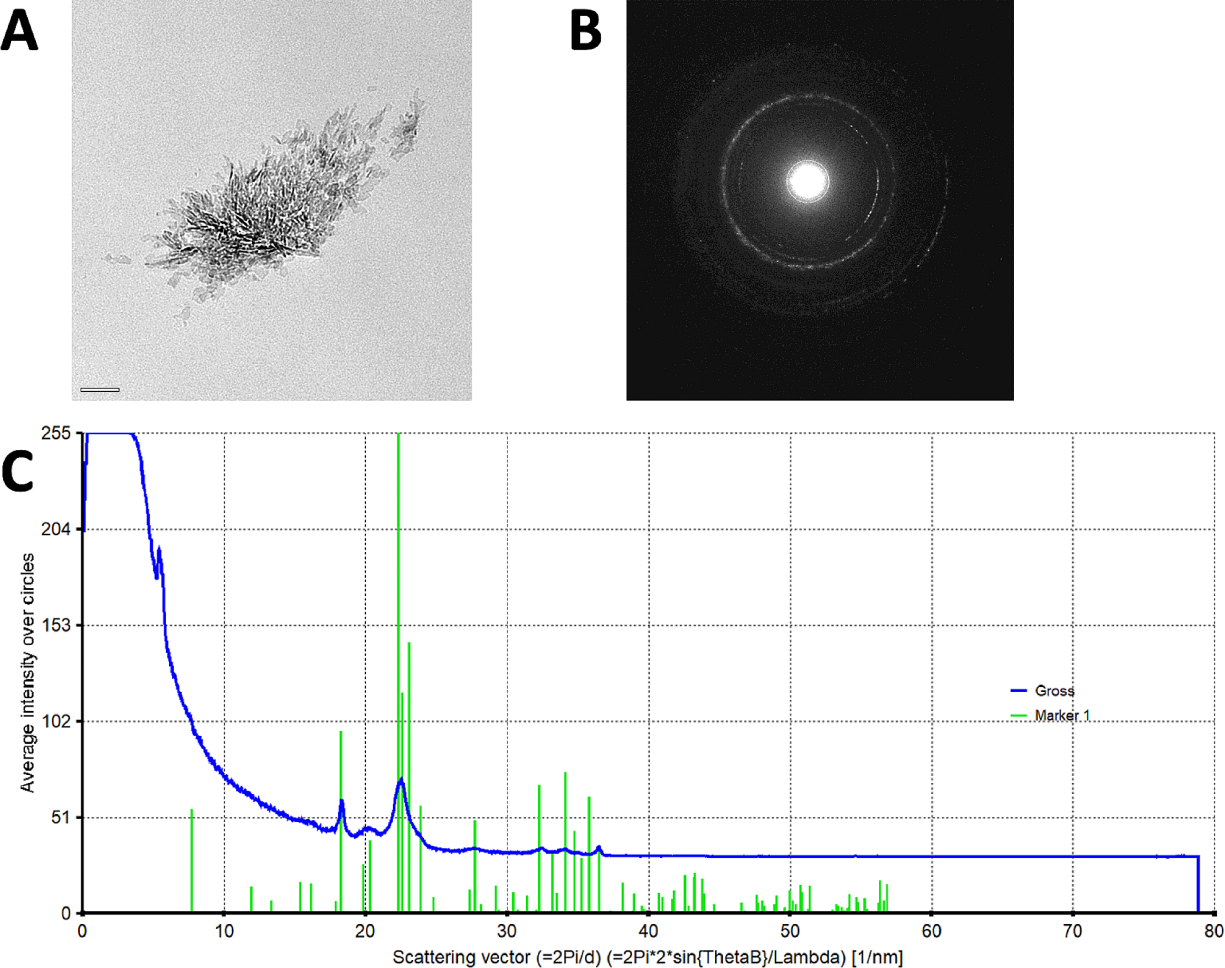
Electron crystallography of crystal growth in the Lentis IOL polymer following 20 h exposure to 14 mM Na_2_HPO_4_. **a**. Transmission electron microscopy demonstrating crystal growth. **b**. Crystal electron diffraction pattern, used to identify the specific chemical composition and crystal structure in the calcium phosphate crystal present. **c**. The very high match between the crystals’ electron diffraction pattern (gross, blue line) and the database electron diffraction pattern for hydroxyapatite (marker 1, green lines) confirms that the crystal is made of hydroxyapatite. Na_2_HPO_4_: Disodium hydrogen phosphate

Following electrophoresis, the IOLs were rinsed with distilled water and the presence of calcium phosphate crystals within the IOL polymer evaluated by 7 specific methods according to an established protocol used for explanted calcified IOLs (Fig. 1C) [1, 2, 6, 9, 10, 20, 30, 31]. Firstly, gross light microscopy was performed and overview images were obtained. Histologic staining with alizarin red was used to detect superficial calcium phosphate deposits, confirming that ion exchange took place (Figs. 2 and 3). Subsequently IOLs were cut in half, and one half stained with the von Kossa method to identify deposits below the IOL polymer surface in 5 μm vertical cross-sections of the IOL (Figs. 2 and 3). The other half of the IOL was sent to the Max Planck Institute for polymer research in Mainz (Germany) to perform scanning electron microscopy (SEM) and electron diffraction x-ray spectroscopy (EDX) analyses on any detected crystals (Fig. 4). Transmission electron microscopy (TEM) with electron diffraction (ED) were performed following EDX, allowing definitive identification of the specific calcium phosphate crystal present (Fig. 5) [10].

### Quantification of IOL calcification

**Fig. 6:**
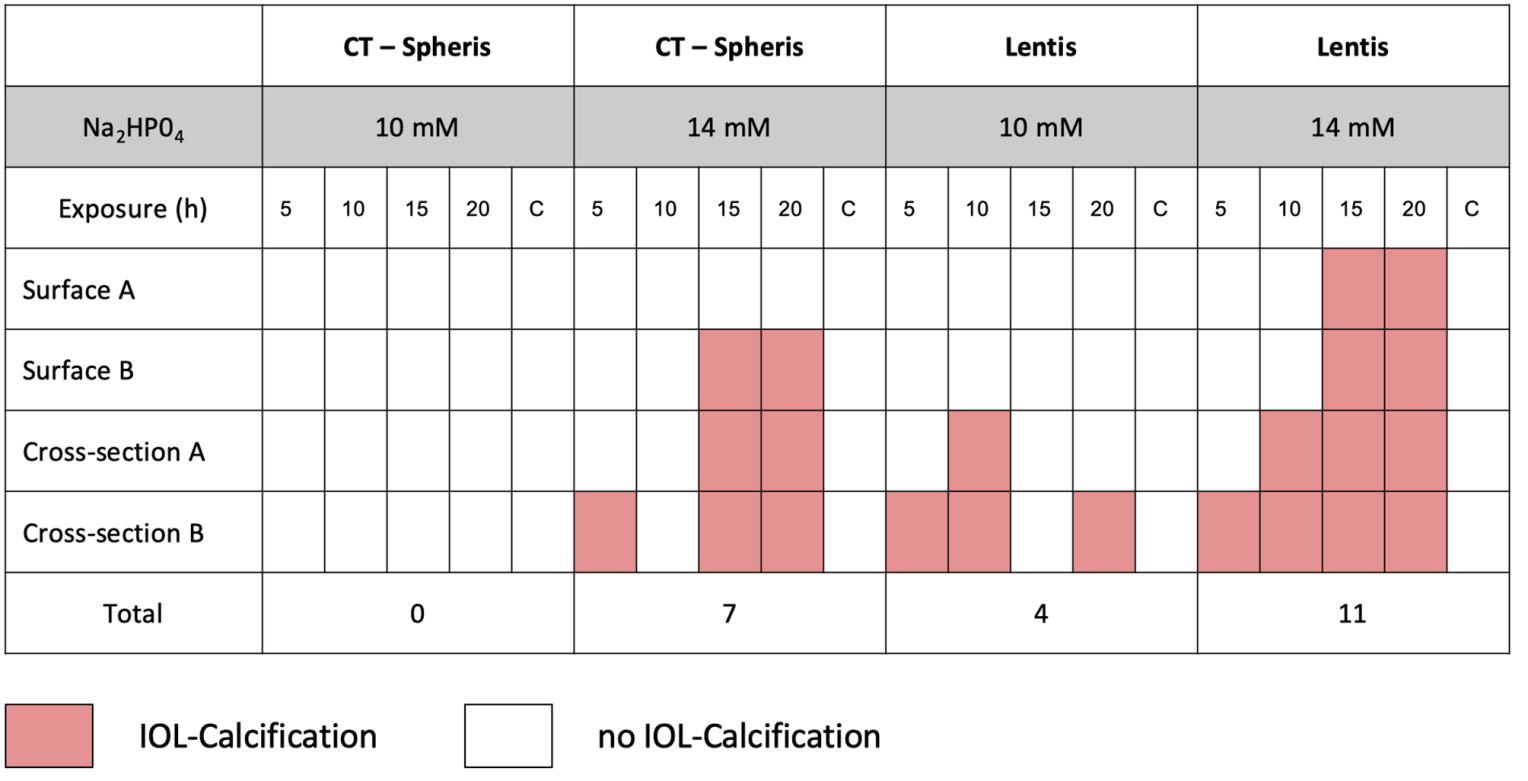
Qualitative analysis of IOL calcification based on SEM analyses. Both surfaces and sides of the IOL’s cross-sections were analyzed for crystal growth (present/not present) after every duration of exposure (5, 10, 15 and 20 h). C: control IOL, h: hours, mM: millimolar

**Fig. 7:**
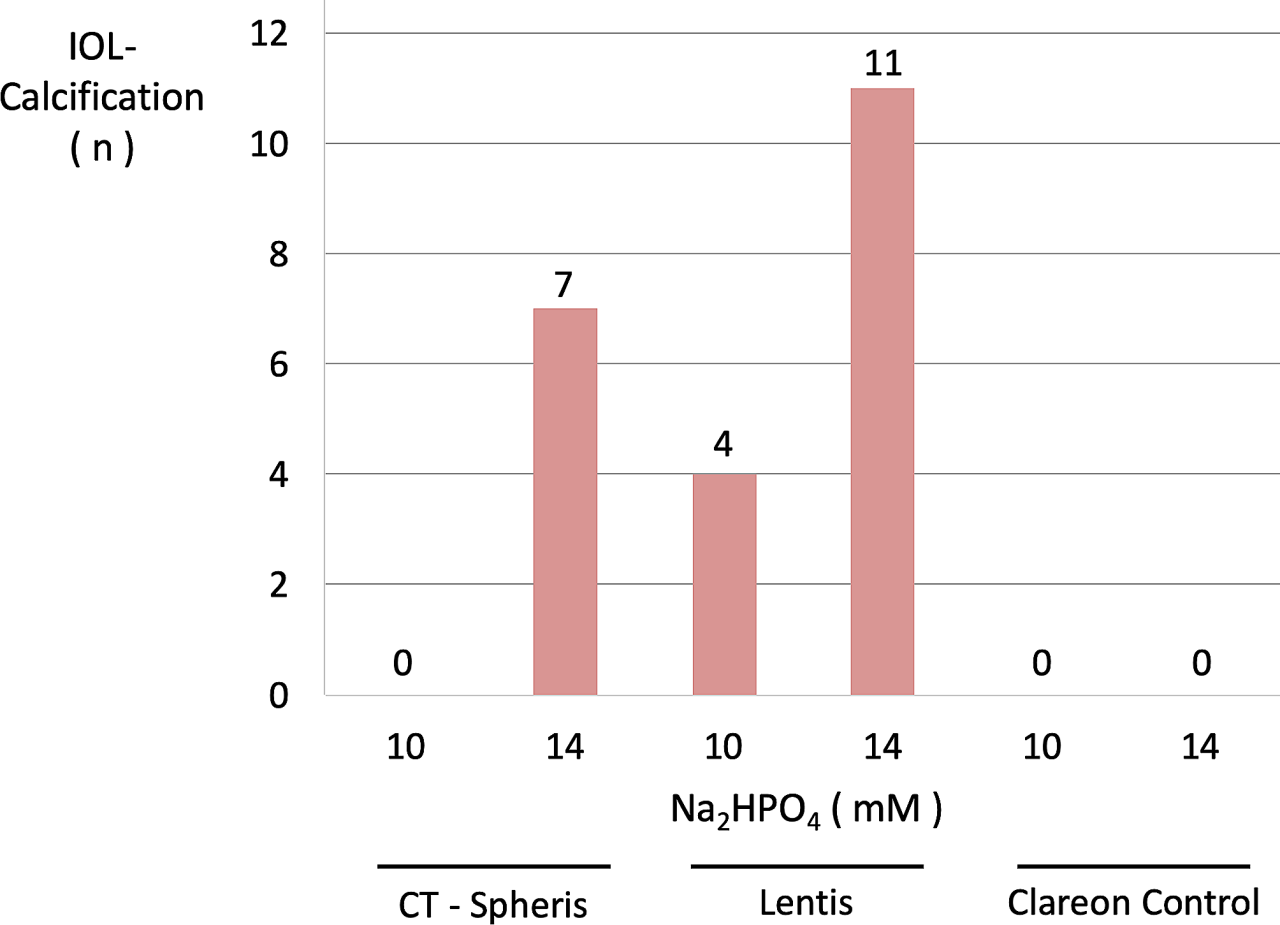
Total number of documented IOL calcifications in SEM analyses (present/not present) for every IOL model and concentration tested (Fig. 6), illustrating the clear association between Na_2_HPO_4_ concentration and IOL calcification. Na_2_HPO_4_: Disodium hydrogen phosphate

To quantify the extent of IOL calcification a semi-quantitative approach was developed. This approach entailed analysis of both IOL surfaces and the cross-sections of every IOL using SEM images for the presence of calcium phosphate crystals (calcification present or not-present). Data from both surfaces and cross-sections of every IOL were summarized and the extent of IOL calcification documented (Figs. 6 and 7).

## Results

### IOL calcification analysis

**Fig. 8:**
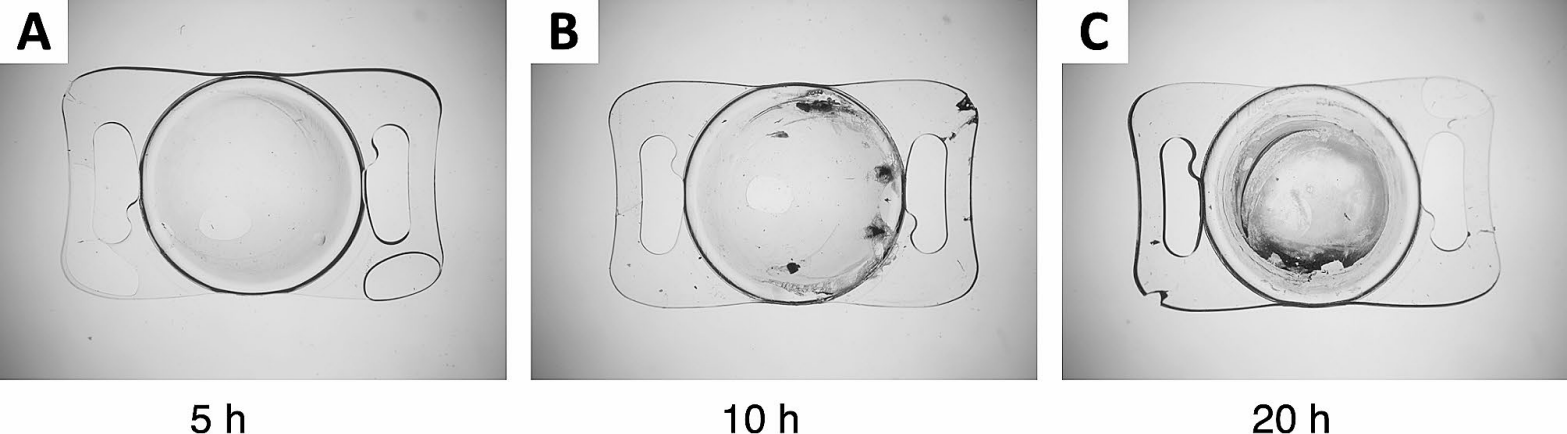
Lentis IOLs following 5 **(a)**, 10 **(b)** and 20 h **(c)** of exposure to 14 mM Na_2_HPO_4_, illustrating the association between degree of calcification and duration of exposure. Na_2_HPO_4_: Disodium hydrogen phosphate

Exposure of hydrophilic CT Spheris IOLs to 10 mM Na_2_HPO_4_ for up to 20 h did not lead to IOL calcification. In contrast, in hydrophilic Lentis IOLs exposed for 20 h traces of calcium phosphate were detected using von Kossa staining and SEM (Fig. 2). Exposure of both IOL types to 14 mM Na_2_HPO_4_ lead to IOL calcification, more so in IOLs exposed for 20 h compared with 5 h and controls (Figs. 3 and 8). EDX microanalyses performed for all crystals detected in IOL cross-sections demonstrated the presence of calcium and phosphorus (Fig. 4). TEM and ED pattern analyses identified the calcium phosphate salt as hydroxyapatite (Fig. 5).

Crystal deposits were observed on the surface of the Clareon control IOL in the area of the IOL holders. Due to the hydrophobicity of the Clareon material, the ions dissolved in the aqueous medium cannot diffuse the material and crystallize into hydroxyapatite. However, sodium salts of phosphoric acid can be formed on the cathode side or calcium salts of hydrochloric acid on the anode side. These salt precipitates can be observed on the surface (Fig. 3). Furthermore, as no traces of calcium-phosphate were detected in alizarin red -, von Kossa staining or SEM (Fig. 3), it can safely be concluded that these are not traces of IOL calcification.

### Comparison of IOLs exposed to 10 mM vs. 14 mM Na_2_HPO_4_

**Fig. 9:**
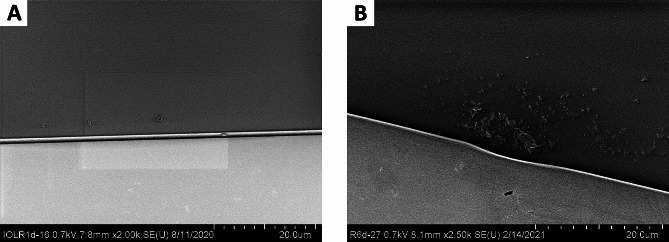
Comparison of crystal growth in SEM magnification of the CT Spheris IOL following 20 h exposure to 10 mM and 14 mM Na_2_HPO_4_. **a**. Magnification of CT Spheris IOL cross-section following 20 h exposure to 10 mM Na_2_HPO_4_, documenting no calcium phosphate crystal growth. **b**. Magnification of CT Spheris IOL cross-section following 20 h exposure to 14 mM Na_2_HPO_4_, documenting calcium phosphate crystal growth and demonstrating that crystal growth only occurred following exposure to the elevated Na_2_HPO_4_ concentration. SEM: Scanning electron microscopy, Na_2_HPO_4_: Disodium hydrogen phosphate

IOL calcification occurred in 0 of 4 CT Spheris and 3 of 4 Lentis IOLs following exposure to 10 mM Na_2_HPO_4_, compared with 3 of 4 and 4 of 4 calcified IOLs following exposure to 14 mM Na_2_HPO_4_, respectively (Fig. 6). Therefore, in CT Spheris IOLs calcification occurred only following exposure to 14 mM Na_2_HPO_4_ (Fig. 9). In Lentis IOLs calcification was more pronounced in lenses exposed to 14 mM Na_2_HPO_4_ compared with 10 mM Na_2_HPO_4_ (Figs. 2 and 3) and in lenses exposed for 20 h compared with 5 h and controls, illustrating a clear correlation between increased phosphate concentration, duration of exposure and IOL calcification, confirming duration of exposure as an additional risk factor for IOL calcification (Fig. 8).

### Semi-quantitative analysis of IOL calcification

A semi-quantitative approach, based on calcification of both IOL surfaces and IOL cross-sections, reflects differences in the extent of calcification between CT Spheris and Lentis IOLs, as calcification occurred in 0 CT Spheris and only 4 Lentis IOLs following exposure to 10 mM Na_2_HPO_4_, compared with 7 and 11 positive analyses following exposure to 14 mM Na_2_HPO_4_. Therefore, the qualitative aspects (present /not present) in the SEM analyses of all IOL surfaces and their cross-sections illustrate the clear association between increases in phosphate concentrations and IOL calcification (Figs. 6 and 7). No IOL calcification occurred in control IOLs (Figs. 3, 6 and 7).

## Discussion

The present analyses investigated the potential pathogenetic impact of phosphate concentrations typically found in aqueous humor of patients with diabetes on calcification of hydrophilic IOLs. A clear association between phosphate concentration in diabetic aqueous solution and IOL calcification was confirmed. An equally clear association between IOL calcification and duration of phosphate exposure was demonstrated, suggesting that both increased phosphate concentrations and duration of exposure are typical risk factors of IOL calcification.

These findings are in line with the fact that IOL calcification has been related to diabetes in numerous case studies [14, 15, 17, 22–26]. As mentioned, increased phosphate concentrations were found in aqueous humor of patients with diabetes, with the highest concentrations in patients suffering from proliferative diabetic retinopathy [15]. The implied correlation between the degree of diabetic retinopathy and aqueous humor phosphate concentration supports one of the theories behind the pathogenesis of IOL calcification, as proliferative diabetic retinopathy is known to cause a breakdown in blood-aqueous barrier [14]. This would explain why diabetic patients suffer from significant changes in aqueous humor composition, a potential causal mechanism behind IOL calcification, and are therefore at a higher risk to develop IOL calcification. Furthermore, whilst the physiologic aqueous humor already represents a solution supersaturated with calcium and phosphate, the increased phosphate concentrations associated with diabetes and our findings suggest that the increase in phosphate concentration in aqueous humor of diabetic patients are a main driving force behind IOL calcification. The increased aqueous humor phosphate concentration surrounding the IOL accelerates ion diffusion and IOL calcification.

The impact of the local microenvironment surrounding implanted IOLs and its pathogenetic influence on IOL calcification is strengthened by variations of IOL calcification found in supplementary and capsular IOLs implanted in the same eye [10]. This theory is further supported by the hypothesis that changes in aqueous humor pH value following ocular glaucoma surgery, representing a change in microenvironment, promote calcium salt precipitation by interruption of the blood-aqueous barrier [32]. As opposed to exclusive metabolic changes in aqueous humor, changes in the intraocular convective motions of the aqueous humor are considered an alternative mechanism contributing to IOL calcification [33]. In addition to changes in aqueous humor and comorbidities, surgical procedures performed subsequent to IOL implantation are an additional frequent cause for IOL calcification [6, 13, 18–22].

The molecular mechanisms underlying IOL calcification are as yet poorly understood. Several factors likely contribute to IOL calcification. Firstly, calcification occurs when the solubility product of ions in solution has been exceeded, causing precipitation to occur where the ions form a salt [34].

In the case of implanted IOLs, as the aqueous humor is supersaturated with calcium and phosphate ions, when the calcium and phosphate ions reach saturation in the IOL polymer, precipitation and therefore calcium phosphate salt formation occurs [1, 2]. The precipitated calcium phosphate salts act as nuclei for further crystal formation in the IOL polymer [29, 35, 36]. The IOL water content is thought to play a major role, explaining why hydrophobic IOLs, typically made of Poly-methyl methacrylate (PMMA), do not show calcification as their water content is significantly lower, usually only between 1–4% [1, 2, 37]. In contrast, hydrophilic acrylic IOL material has a much higher water content and is usually a poly-2-hydroxylethyl methacrylate (PHEMA) polymer or a copolymer of 2-hydroxyethyl methacrylate with methyl methacrylate, containing multiple carboxylic and hydroxyl groups.

These groups, present in the hydrophilic IOL polymer, contribute to a strongly negative charge, causing positively charged calcium ions to complex, increasing the local ion concentration and thereby facilitating salt precipitation in the IOL polymer [1, 38]. In addition, the increased number of IOL hydroxyl groups of the polyacrylic materials may facilitate nucleation and crystal growth, either through ionization or incorporation of phosphate groups [1]. Most of the currently available hydrophilic acrylic PHEMA IOLs have a water content ranging from 18 to 38%. Higher extent of hydration leads to higher degree of ionization of the polymeric functional groups, thus promoting calcification through the formation of complexes with ionized calcium [1, 38].

Drimtzias and colleagues showed in an in vitro model that calcification occurred when the IOL was exposed to a solution supersaturated with calcium phosphate crystal phases [31]. The mineral formation started within the IOL, with the front of calcium phosphate crystallites advancing toward the anterior IOL hydrophilic surface owing to ion diffusion [1, 31]. These results suggest that IOL calcification is initiated in the interior of hydrophilic IOLs and then advances towards the outside. The present findings support this theory, as Lentis IOLs exposed to 14 mM Na_2_HPO_4_ for 15 h showed bumps due to subsurface deposits on the IOLs surface. EDX microanalysis identified the mineral underlying the bumps as calcium phosphate. SEM of the IOL interior detected crystal growth in all Lentis IOL cross-sections exposed to 14 mM Na_2_HPO_4_, meaning 5 h of exposure were sufficient for crystal growth to occur in the interior. These results strengthen the growth pattern hypothesis as SEM documented crystal growth in all IOL cross-sections, whereas 15 h of exposure were necessary for superficial deposits and bumps in the surface to occur.

TEM and ED, techniques still new to the field, were used to characterize crystals, identifying their specific chemical composition and structure, in the IOLs tested. Hydroxyapatite, the salt also present in explanted calcified IOLs, was identified as the specific calcium phosphate phase present [2, 10]. Consequently, it is safe to conclude that the electrophoresis model used in the present analyses and modified to test the influence of the diabetic metabolic state on IOL calcification, mimics in vivo IOL calcification, an ideal basis for further evaluation of risk factors and mechanisms behind IOL calcification [30]. This is true even though not all analyses confirm the clear association between diabetes and an increased risk of IOL calcification, underscoring remaining open questions and the need to better understand both the true influence of patient individual factors and whether IOL calcification can be definitely linked to certain circumstances [39].

Given that both the CT Spheris and the Lentis IOLs are hydrophilic acrylic IOLs with a water content of 25% and similar hydrophobic surface properties, the observed differences between the two IOLs may be consequent to individual material properties. Differences seen between the two IOL models, obviously influencing the risk of IOL calcification, may be a consequence of these individual material properties. Irrespective of IOL model, the hydrophobic surface on the hydrophilic acrylic intraocular IOLs tested in the present analyses does not seem to disrupt the process of calcification in these IOLs. This is supported by several clinical case series describing calcification in hydrophilic IOLs with hydrophobic surface properties [1, 3, 39–42]. The hydrophobic surface coating does not seem to compromise ion diffusion and therefore IOL calcification, underscoring the need to include uncoated IOLs as additional controls in further analyses.

Real world evidence demonstrates that some IOL models are more susceptible to calcification, a likely consequence of individual IOL properties. The experimental model used in the present analyses offers a set-up for screening of different IOL models regarding their individual disposition to develop calcification especially in diabetic eyes. Given the good biocompatibility, low cost and ease of implantation of hydrophilic IOLs and the fact that IOL calcification is relatively rare, a less attractive conclusion would be to no longer use hydrophilic IOLs in diabetic patients, however, hydrophobic IOLs may also develop opacities, mainly glistenings [43, 44]. These considerations may increase in clinical relevance as both the increasing life expectancy will increase cataract incidence and the need for IOL implantation and the number of patients suffering from diabetes is constantly rising, expected to grow from 285 to 489 million by 2030 [45]. Furthermore, diabetes in itself promotes cataract formation necessitating cataract surgery and IOL implantation prematurely to most other patients [46]. This implies that the average dwell time of an IOL in the eye will be proportionally increased in diabetics. As duration of exposure was an additional risk factor correlating with the degree of IOL calcification, the necessity to minimize all other risk factors is emphasized. In addition, the potential duration of stay of implanted IOLs in the eye will likely also increase due to higher life expectancy in general and as cataract surgery with IOL implantation is also performed in treatment of infantile cataract, the potential dwell time will likely increase to several decades [47]. Finally, estimates assume that the realistic incidence of IOL calcification is higher than documented as solely IOL explantations are recorded, not considering patients in whom the risk of IOL exchange is too high and the procedure therefore not performed [48].

Strengths of the present analyses are the clear correlation between likely risk factors of IOL calcification in diabetic patients and calcification itself, the attractiveness of this model to improve the understanding of pathophysiological mechanisms behind IOL calcification as well as the clinical potential to screen different IOL models regarding their individual risk for calcification. Furthermore, future applications lie in the analysis and testing of IOL materials and prototypes still in development, optimizing these materials for their risk of IOL calcification. A potential weakness and limitation is the limited number of IOLs tested, preventing a statistical interpretation of the data, a direct consequence of the extensive and time-consuming methods included in the IOL analysis protocol (Fig. 1C). On the other hand, as previous models needed 18 months to induce IOL calcification, our electrophoresis model with only a 20 h duration represents an important step forward in the ability to model these processes [30, 31].

The electrophoresis model utilizes harsh conditions to induce calcification in a short time period rather than precisely replicating in vivo conditions. However, as shown by Britz et al., the verification of hydroxyapatite, the calcium phosphate salt also found in both alternative calcification models used to simulate calcification and in IOLs explanted in vivo, confirms that our model mimics the process of in vivo IOL calcification, usually spanning many years, within a few hours [30, 31].

## Conclusions

In conclusion, a correlation between increased phosphate concentrations as well as the duration of exposure and IOL calcification was demonstrated for both CT Spheris and Lentis IOLs, clearly showing that both factors contribute to increased IOL calcification. Furthermore, differences between IOL models regarding susceptibility to IOL calcification were shown, a potential consequence of IOL’s individual material properties. Altogether, whilst some overarching risk factors increase calcification, IOLs still possess individual characteristics that may facilitate and therefore influence calcification. Further research into risk factors and underlying pathomechanisms leading to IOL calcification is mandatory to allow individual properties of IOL polymers to be modified to minimize IOLs individual risk of calcification and thereby reduce calcification even in the presence of risk factors.

## Data Availability

No datasets were generated or analysed during the current study.

## Abbreviations

CaCl_2_: Calcium chloride
ED: Electron diffraction
EDX: Energy dispersive x-ray spectroscopy
IOL: Intraocular lens
Na_2_HPO_4_: Disodium hydrogen phosphate
PHEMA: Poly-2-hydroxylethyl methacrylate
SEM: Scanning electron microscopy
TEM: Transmission electron microscopy
